# Videogrammetric Verification of Accuracy of Wearable Sensors Used in Kiteboarding

**DOI:** 10.3390/s21248353

**Published:** 2021-12-14

**Authors:** Marián Marčiš, Marek Fraštia, Andrej Hideghéty, Peter Paulík

**Affiliations:** 1 Department of Surveying, Faculty of Civil Engineering, Slovak University of Technology in Bratislava, 81005 Bratislava, Slovakia; marek.frastia@stuba.sk (M.F.); andrej.hideghety@stuba.sk (A.H.); 2 Department of Concrete Structures and Bridges, Faculty of Civil Engineering, Slovak University of Technology in Bratislava, 81005 Bratislava, Slovakia; peter.paulik@stuba.sk

**Keywords:** kiteboarding, videogrammetry, jump height measurement, wearable sensors

## Abstract

Owing to the combination of windsurfing, snowboarding, wakeboarding, and paragliding, kiteboarding has gained an enormous number of fans worldwide. Enthusiasts compete to achieve the maximum height and length of jumps, speed, or total distance travelled. Several commercially available systems have been developed to measure these parameters. However, practice shows that the accuracy of the implemented sensors is debatable. In this study, we examined the accuracy of jump heights determined by sensors WOO2 and WOO3, and the Surfr app installed on an Apple iPhone SE 2016, compared to a combination of videogrammetric and geodetic measurements. These measurements were performed using four cameras located on the shore of the Danube River at Šamorín, Slovakia. The videogrammetrically-determined accuracy of jump heights was 0.03–0.09 m. This can be considered a reference for comparing the accuracy of off-the-shelf systems. The results show that all of the systems compared tend to overestimate jump heights, including an increase in error with increasing jump height. For jumps over 5 m, the deviations reached more than 20% of the actual jump height.

## 1. Introduction

Kiteboarding (or kitesurfing, depending on the region in which the term is used) is a relatively young adrenaline sport [1,2]. Its origins date back to 1977 when Dutchman Gijsbertus Adrianus Panhuise patented a water sport using a surfboard and a parachute device to manipulate wind force [3]. However, the development of an inflatable kite by the Legaignoux brothers enabled the practical public use of kiteboarding [4]. The first competitive event was held in Maui in 1998.

Competitions currently take place in disciplines such as wave-riding, freestyle, or course racing [5]. Results are highly dependent on weather conditions and the athlete’s equipment, physical condition, and experience. The greatest risk of injury occurs in competitive kiteboarding [6]. In addition, the inability to detach the kite from the harness during uncontrollable wind drift is the most common cause of death, often even among experienced instructors.

Athletes use different types of sensors to analyse their performance. These are usually a combination of accelerometers, magnetometers, and gyroscopes, sometimes supplemented by GPS units. There are currently an enormous number of commercially available systems for the analysis of the movement of athletes [7]. Within kiteboarding, the most used systems include WOO (woosports.com (accessed on 10 July 2021)), PIQ (piq.com (accessed on 10 July 2021)), Xensr (xensr.com (accessed on 10 July 2021)), or the Surfr application (thesurfr.app (accessed on 10 July 2021)), which require installation on a smartphone equipped with an accelerometer and gyroscope. The accuracy of the determined jump parameters depends on: (1) the accuracy of the sensors used and the method of their initialisation and (2) the current conditions of the jump, its total height, and the duration of the climb. In principle, the larger the jump, the lower the accuracy of the measurement because of the accumulation of systematic errors [8].

An important milestone in the field of competitive kiteboarding was the release of the current version of the WOO3 system, which is connected to the Leaderboards community service. At the time of the experiment, Maarten Haeger from Netherlands was the ranking leader, with a jump of 34.8 m [9]. However, the accuracy of the recorded height remains questionable despite the constant efforts of the kiteboarding community to compare results from different systems. Comparisons [10,11,12] have shown that WOO systems tend to overestimate jump height. In general, differences between systems can be 1 m or more. Thus far, however, no study has been performed that compares the results from these sensors to a more accurate reference measurement method such as videogrammetry.

Videogrammetry has long been successfully used in the analyses of various sports. It is based on photogrammetric principles and uses several cameras to allow the evaluation of 3D coordinates based on 2D measurements of image coordinates. While photogrammetry is normally used only for static scenes, videogrammetry can be used to analyse dynamic phenomena with the help of synchronised cameras. The main motivation of most applications is to minimise the causes of various types of injuries or to increase the performance of athletes based on a detailed motion analysis. Typical examples of the use of videogrammetry in sports are: determining the speed of collisions of American football players, either to improve the efficiency and safety of helmets [13,14] or verify the accuracy of the videogrammetry [15]; analysing posture abnormalities of badminton players to reduce the risk of various injuries [16]; analysing the arm kinematics of injured and uninjured archers [17]. The accuracy of videogrammetric measurements depends on several factors [18,19] such as:

- geometric resolution of the cameras used,
- locations of the cameras in relation to the monitored object,
- video sync accuracy,
- methods for measuring image coordinates (manually or automatically),
- method of signalling the evaluated points,
- number of projections of the evaluated points (minimum two),
- video framerate,
- shutter speeds for individual images,
- lighting conditions, and
- speed of the captured movement.

In addition, the calibration of the camera (i.e., the determination of the interior orientation parameters) should be an indispensable part of any photogrammetric processing. Knowledge of these elements makes it possible to accurately mathematically model the path of the rays that refract after passing through the lens, thus increasing the accuracy of photogrammetric processing. There has been considerable progress in the field of self-calibration [20], where a camera can be calibrated directly on images of the object itself. However, it is appropriate to perform calibration for videogrammetric applications separately under ideal conditions that are usually absent in the analysed moving scene. Additionally, photogrammetry is often associated with geodetic measurements to determine the coordinates of ground control points (GCPs), which allow the transformation of photogrammetrically-determined 3D model coordinates into a 3D reference coordinate system. This ensures the correct translation, rotation, and scaling to the coordinate system in which the experiment needs to be analysed. GCPs are also useful in determining the relative and exterior orientation (the position and rotation of the camera at the time of exposure) of the images, thus minimising possible deformations of the camera network. The measurement of these points can be performed with millimetre accuracy using surveying equipment, such as a total station [21].

The main advantage of videogrammetry in comparison with other methods of measuring dynamic phenomena, such as wearable sensors, is the ability to capture the state of the object as a whole. Once the video has been captured, the processor can determine whether only artificially signalled target points will be evaluated or also other natural elements of the object. The video provides additional information about the state of the object at the analysed moment, making it easier to identify the causes of possible discrepancies in the processing results. The disadvantages of videogrammetry include decreasing accuracy with increasing distance from the camera and increased demands on the quality of the equipment, depending on the speed of the analysed motion [19]. Overall, however, photogrammetry, including videogrammetry, is a low-cost and accurate measurement method.

Given the advantages of videogrammetry, we set two specific goals for this study: (1) to determine the accuracy of selected wearable sensors at various jump heights and (2) to investigate the assertion that the selected sensors overestimate jump height.

## 2. Materials and Methods

**Figure 1:**
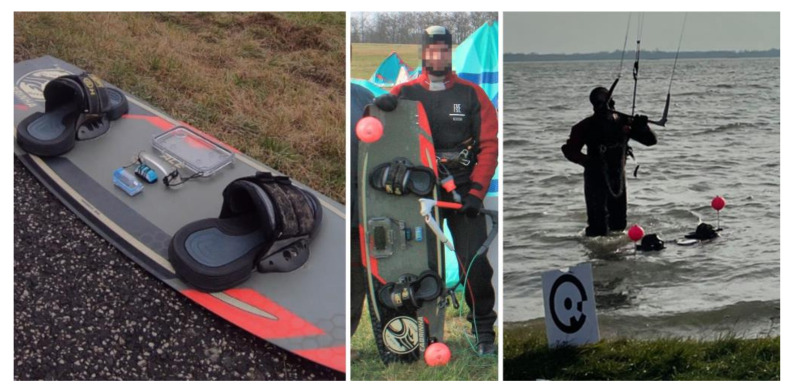
2015 Cabrina Ace twintip, including WOO2 and WOO3 sensors, iPhone SE smartphone, and polystyrene spherical targets for videogrammetry.

The experiment was conducted on 6 December 2020 at the Hrušov water reservoir on the Danube River, near the city of Šamorín, 10 km from the capital city of Bratislava, Slovakia. The purpose of the videogrammetric measurement was to determine jump heights. For this purpose, spherical polystyrene targets were placed on a 2015 Cabrina Ace twintip (137 × 41 cm). This board was equipped with WOO2 and WOO3 sensors, along with an attached Apple iPhone SE 2016 smartphone (model A1723) (Figure 1). The kiter used a 12 m^2^ 2020 North Orbit kite. This equipment has been recommended for Big Air/Freeride disciplines, wherein the goal is to achieve the greatest possible jump height.

**Figure 2:**
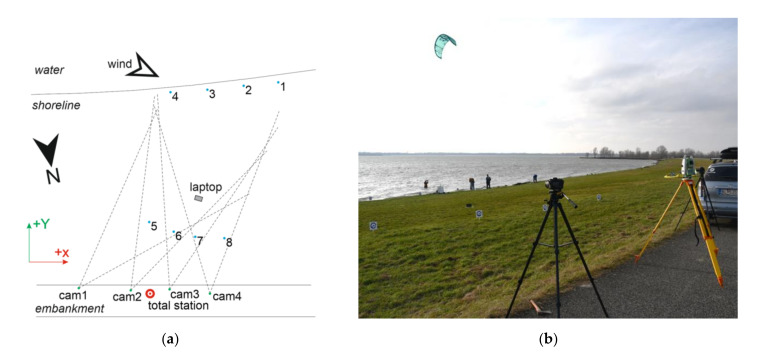
(**a**) Plan of the placement of cameras and GCPs; (**b**) Shore conditions during the experiment.

Four Nikon D7500 DSLR cameras with AFS Nikkor 24 mm 1:1.8G ED lenses on tripods were placed on the raised bank of the reservoir and used for videogrammetric measurements (Figure 2a). Eight coded GCP targets were stabilised between the cameras and the shore. Their 3D coordinates were determined using the Leica TS06 plus total station (Figure 2b). The targets were printed on 30 × 30 cm high impact polystyrene (HIPS) boards and fixed to the ground using wooden pegs. The distance between adjacent cameras was approximately 6 m, and the shore was approximately 30 m from the cameras. The relative positions of the targets and cameras were chosen to observe at least six GCPs from each camera position.

**Figure 3:**
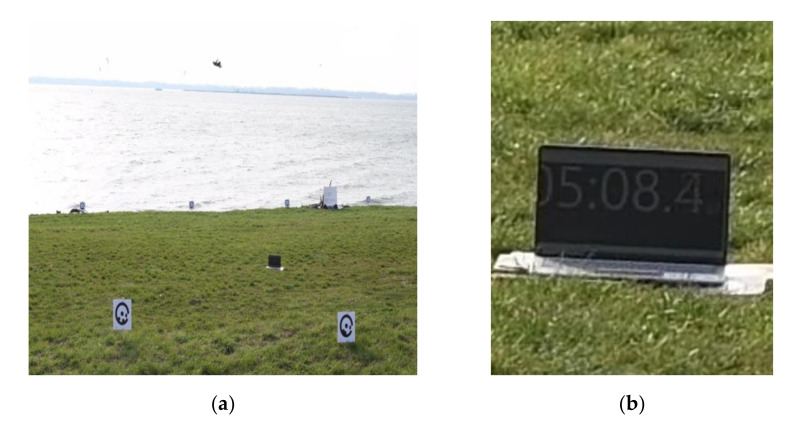
(**a**) Sample field of view from camera 3; (**b**) Detail with the laptop timer.

The video was recorded at 4K resolution with a frequency of 30 fps. We captured 4 × 24 GB of data from all cameras (approximately 4 × 30 min of video). The synchronisation of individual video recordings was ensured by means of a timer running on a laptop screen in the field of view of all cameras (Figure 3a,b). The cameras and wearable sensors had differing starting times. Therefore, it was necessary to determine the time discrepancy between the two measurement methods. This was accomplished by visual comparison of the data based on the size of the jumps and the time differences between them.

The kiter attempted to perform the jumps as close to the shore as possible. The distance between the cameras and the place of the jump was approximately 45–120 m. The primary benefit of using four cameras was the extended lateral coverage of the water surface, as the jumper had to be visible from at least two camera positions for evaluation. In addition, each additional projection increased the reliability of the evaluated 3D coordinates of the observed marks. There were two motivations for placing two contrasting marks on the twintip board: (1) to calculate the average board position based on the positions of each mark because the sensors were placed in the middle of the board, and (2) to ensure that it was always possible to evaluate at least one mark if the other was hidden behind an obstacle.

## 3. Videogrammetric Processing

The bulk of the processing was performed using the photogrammetric software Agisoft Metashape Professional (agisoft.com (accessed on 21 January 2021)), which also contains a 4D module. Although the software is primarily used for the automated generation of point clouds from textured surfaces using computer vision techniques, it also allows automatic and manual measurement of coded or circular points.

The structure from motion (SfM) analysis process is based on the relative orientations of captured images. However, because of the specific positions of the cameras relative to the scene being captured, the SfM process would produce unreliable results for this study due to the lack of tie points detected in texture [22,23]. Therefore, coded targets with geodetically measured 3D coordinates served to define the local coordinate system and refine the elements of the relative and exterior orientation of the cameras during the bundle adjustment. This process incorporated an attempt to minimise so-called reprojection errors, that is, the deviation between the measured position of a point on the image and its reversed projection into the image from its resulting 3D position in the scene.

### 3.1. Camera Calibration

Calibration of the camera system is integral for the accurate determination of the parameters of interior orientation (the camera and lens parameters) in photogrammetric processing. Calibration was performed individually for each camera, although four identical cameras and lenses were used. Experience has shown that, because of possible manufacturing variations, highly accurate photogrammetric applications require the separate calibration of each system. Moreover, the distance to the calibration point field should be approximately equal to the distance to the object captured for analysis. Due to refocusing, the elements of interior orientation (especially the focal length) can also change.

**Figure 4:**
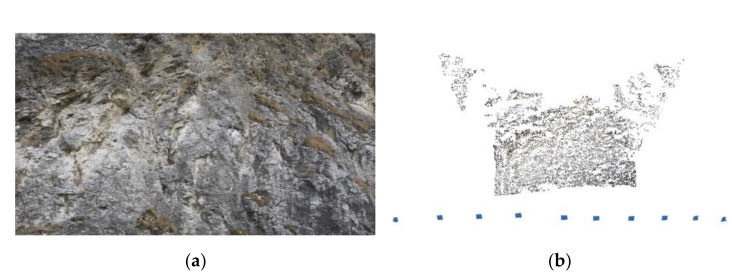
(**a**) Preview image of the rock from the calibration video; (**b**) resulting 3D scene with tie points and cameras.

A short video sequence of a rock slope was used for calibration (Figure 4a). The calculation in the Agisoft Metashape was based on the principles of self-calibration in the SfM process (Figure 4b).

Interior orientation calibration results for each of the cameras used in the study are shown in Table 1, where f represents the principal distance (focal length); w and h are the dimensions of the image sensor in mm and pixels; xp and yp are the image coordinates of the principal point (perpendicular projection of the projection centre into the image plane); K1, K2, and K3 are the coefficients of radial distortion of the lens; P1 and P2 are the coefficients of tangential distortion of the lens; P is the pixel size of the image sensor in mm.

The elements of interior orientation in Table 1 differ only minimally between the individual cameras. In empirical testing for this project, the use of individual calibration files did not increase the accuracy of videogrammetric processing because other effects were more significant (discussed in Section 4.2). In the case of using cameras and lenses of the same type, the differences caused by manufacturing variations can potentially be neglected in practice and processing can be simplified by applying only one common calibration file.

Although the Nikon D7500 uses a 23.5 × 15.7 mm (5568 × 3712 pixel) APS-C sensor, only the 16.2 × 9.1 mm image cut-out of this size is used in the 4K video mode (3840 × 2160 pixels) without the need for resampling (pixel size does not change). In combination with the focal length, which also does not change, the field of view is reduced to 70% after switching to the video mode.

### 3.2. D Processing

**Figure 5:**
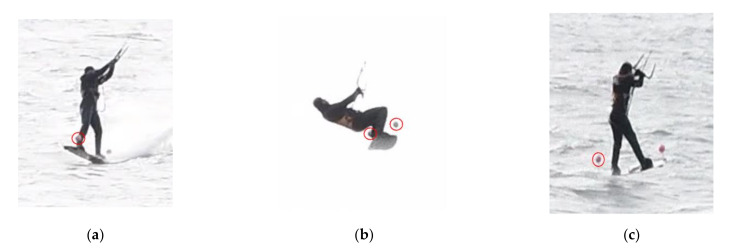
For each of the 20 jumps, (**a**) one spherical target was measured on five images before leaving the water surface; (**b**) both targets were measured on 15–20 images nearest to the highest position in the air, and (**c**) one target was measured on the first image after re-contact with the water surface.

After extracting the images from the videos, it was necessary to sort them according to individual jumps. Visual inspection between adjacent cameras based on the movements of the kiter made it possible to achieve a synchronisation error below 1 fps. Twenty jumps were processed, two of which were selected for detailed analysis of the entire jump trajectory. For the remaining jumps, the spherical targets were evaluated only at the beginning of the jump (Figure 5a), near the maximum height (Figure 5b), and at the end of the jump (Figure 5c). The variable appearance of the spherical targets within the dynamic scene required manual rather than automated measurement. All jumps took place from left to right in the field of view of the cameras.

Photogrammetric processing in Agisoft Metashape consisted of the following steps:

1. loading images into a 4D project,
2. loading the camera calibration protocols and their fixation,
3. manually measuring the GCPs on images in the 1st epoch with the assignment of geodetic reference coordinates,
4. calculating the relative orientation of images (key point limit 40,000, tie point limit 4000, marker accuracy 0.001 m) including transformation into the reference coordinate system,
5. manually measuring the spherical targets on the board,
6. reorienting the project, including targets on the board, and
7. exporting of the results.

Ideally, the tie points should be distributed homogeneously over the entire surface of the images. Because all GCPs were in the lower half of the images (on the ground), step 6 was performed to increase the accuracy of the orientation of the images, including the targets on the board in the upper half of the image.

**Figure 6:**
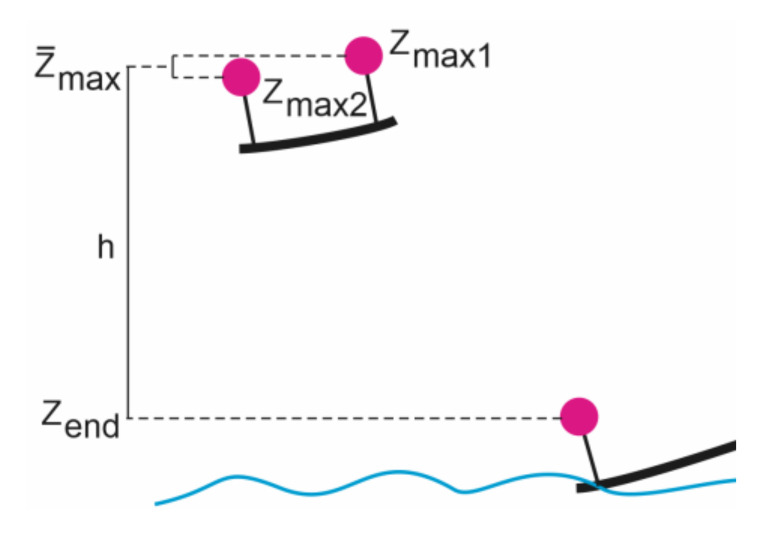
Illustration of jump height calculation from videogrammetrically determined 3D coordinates.

The jump height was calculated from the 3D coordinates as the difference between the highest average Z-coordinate of the pair of targets and the Z-coordinate after re-contact with the water at the end of the jump (Figure 6). The start and end Z-coordinates also served as a control, as the water level was approximately horizontal. The differences between the start and end heights were approximately 20 cm.

## 4. Analysis of Results

### 4.1. Accuracy of Photogrammetric Measurement

The a priori accuracy of a photogrammetric measurement using multiple cameras can be estimated based on the following formula [24]

(1) $$\sigma =\frac{q\cdot D}{f\cdot \sqrt{k}}\cdot P\cdot \sigma_{pix}$$

where *q* represents the quality factor of the camera network, *D* is the distance between the cameras and the object, *f* is the principal distance, *k* is the average number of exposures at each station, *P* is the pixel size of the sensor, and *σ_pix_* is the accuracy of the measured image coordinates. In photogrammetry, the most problematic issue is the accuracy in the direction of the depth of the scene (Y-axis in our study). Improving the accuracy in depth measurements can be achieved by extending the base between adjacent cameras, thereby increasing the ray intersection angle at a specified point. Therefore, in Equation (1), the factor *q* can be replaced by the base ratio *D/B* (*B* is the distance between adjacent cameras) and *k* can be replaced by the number of intersections determining the rays at the measured point. This results in the following formula

(2) $$\sigma =\frac{D}{B}\cdot \frac{D}{f\cdot \sqrt{k}}\cdot P\cdot \sigma_{pix}$$

The longer the base, the higher the a priori accuracy in the position of the point. Similarly, increasing the number of projections of the point will achieve an overdetermination of coordinates and further increase the accuracy.

In Equation (2), the ratio between *D* and *f* is known as image scale *M*. If only one pair of cameras is considered (*k* = 1), Equation (2) ca be transformed into a formula to calculate the a priori accuracy of stereophotogrammetry at the depth of the scene [25]

(3) $$\sigma_{D}=\frac{D}{B}\cdot M\cdot P\cdot \sigma_{pix}$$

The accuracy in the plane parallel to the image plane usually depends only on the accuracy of the measurement of the image coordinates and the size of the pixel after projection on the measured surface (the ground sample distance, GSD). This accuracy can then be calculated according to [25]

(4) $$\sigma_{XZ}=M\cdot P\cdot \sigma_{pix}=GSD\cdot \sigma_{pix}$$

Because the resulting height (*h*) of the jump is calculated as the difference between the two *Z*-coordinates, it is appropriate to adjust Equation (4) as follows

(5) $$\sigma_{h}=\sqrt{2}\cdot GSD\cdot \sigma_{pix}$$

### 4.2. Accuracy of Videogrammetric Measurement

In the videogrammetric measurement of targets on the kiteboard, Equation (2) can be used to determine the a priori accuracy of 3D coordinates, while Equation (5) can be applied to determine the a priori accuracy of the jump height.

**Figure 7:**
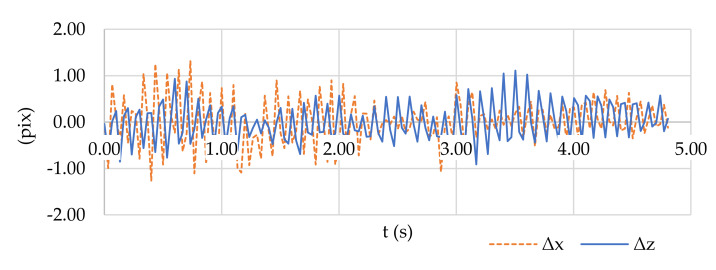
Changes in image coordinates x and z for GCP 5 recorded from camera 1 (coordinates of the coded targets were measured with subpixel accuracy in PhotoModeler software). The vibrations fall within 1 pixel resolution.

However, the critical parameters for calculating accuracy are not only the parameters of the camera network configuration (*D*, *B*, *f*, *k*, *P*) but also the accuracy of measuring the image coordinates *σ_pix_*. Because a moving spherical target with a size of approximately 8 pixels was measured manually, the accuracy of the measurement was approximately 1 pixel. However, during shooting, the cameras were shaken by strong wind gusts. Therefore, the accuracy of the position of the point in the image was also affected by the variable elements of exterior orientation. This issue was corrected during processing. The effect of vibrations on the accuracy of the measurement of the GCPs is shown in Figure 7.

In addition, the resulting 3D position of the moving target may be affected by the inaccuracy of the synchronisation of adjacent cameras. Because the synchronisation accuracy was approximately 1 fps (1/30 s) and the kiter speed was 10 m/s, the inaccuracy in synchronisation should not affect the position of the target by more than 0.3 m, even in the direction of the performed movement. Because the vertical speed of the kiter was near zero at the highest point of the jump, the achieved synchronisation accuracy had a negligible effect on the accuracy of the jump height determination.

**Figure 8:**
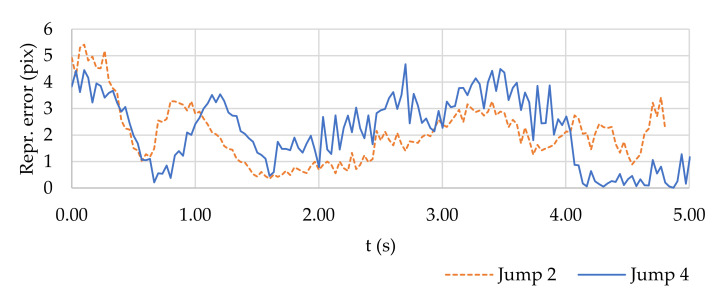
Reprojection error for the spherical target during Jumps 2 and 4. Fine noise component caused by vibrations from wind.

The combination of all the factors influencing the accuracy of the measurements is reflected in the resulting reprojection error of the observed spherical target, as shown in Figure 8.

**Figure 9:**
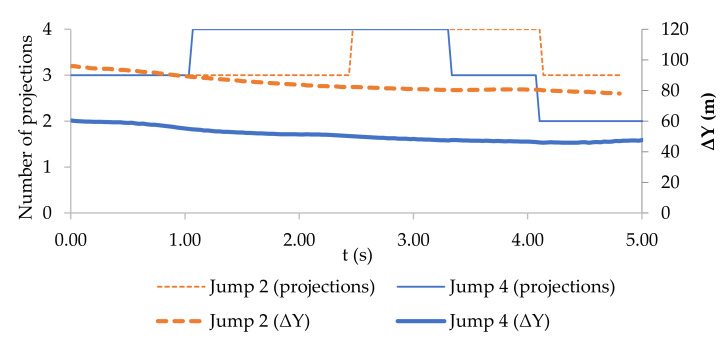
Distance of the moving target from camera 1 during Jumps 2 and 4, and the number of projections of the spherical target during movement in the field of view of the individual cameras.

The course of the reprojection error in Figure 8 shows two selected jumps characterised by specific configurations. Jump 2 was performed at a significantly different distance *D* from the cameras than Jump 4, and the target was monitored using a different number of projections (Figure 9).

The sources of changes in reprojection errors can be considered as a whole:

- camera shake caused by wind (fine noise component),
- change in the number of projections—2 projections produce less reprojection error than an excessive number of projections (e.g., a sharp decrease in the error in Jump 4 after 4 s), and
- sharp movements of the kiter during the ascent in combination with the inaccuracy of the synchronisation of the cameras (significant changes at the beginning of both graphs in Figure 8).

**Figure 10:**
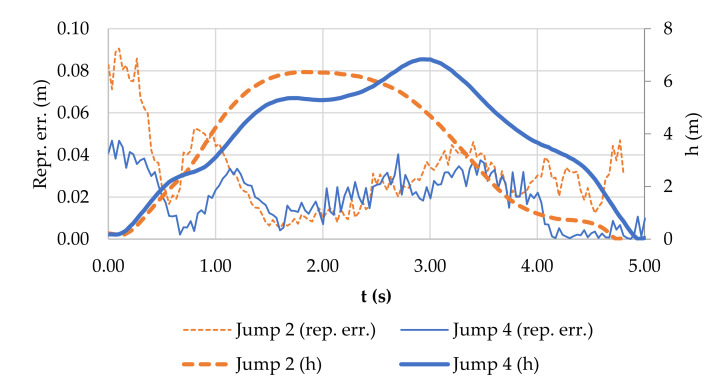
Reprojection error converted to the object plane using the GSD and jump height versus time for Jumps 2 and 4.

When the reprojection error was recalculated using the GSD to the object plane, the metric accuracy was improved in the middle of Jump 4 (Figure 10) despite there being a greater reprojection error in Jump 2 than in Jump 4. The height above the water during Jumps 2 and 4 is shown in Figure 10. The shape of the curve shows that during Jump 4, the kiter tried additional manoeuvres while in the air to maximise the height of the jump.

The root mean square (RMS) of the reprojection error for Jumps 2 and 4 was 2.33 and 2.53 pixels, respectively. If these values are considered to be equivalent to the accuracy of measuring the image coordinates *σ_pix_*, after substituting into Equations (2) and (5), a posteriori accuracy for the 3D coordinates and the maximum jump height may be obtained. The accuracies for Jumps 2 and 4 were *σ* = 0.138 m and *σ* = 0.029 m, respectively, and the maximum jump heights were *σ_h_* = 0.048 m and *σ_h_* = 0.031 m, respectively.

During the trajectory analysis of these two selected jumps, only one spherical target was measured, as the other target was not always visible. The resulting heights in the trajectory for these two jumps may differ slightly from the set of 20 jumps, where heights were calculated as the average of the two targets. The latter situation provided greater accuracy for comparing the results with data from wearable sensors.

### 4.3. Comparison with Wearable Sensors

For the final comparison, 20 jumps were selected, each with heights exceeding 3 m according to the sensors. In addition to the jump heights, Table 2 shows the a priori accuracy of the videogrammetrically determined heights (h_vg_) according to Equation (5). The accuracy of the image coordinates’ measurement in all cases was rounded to three pixels, so only the distance of the observed targets from the camera had a significant effect on the *σ_h_* value. The highest jump (7.3 m) was achieved during Jump 16. Because the jump was completed furthest from the shore with a more suitable wind, the accuracy and distance from the camera were calculated to be 0.089 m and 119.5 m, respectively.

Values h_surfr_, h_WOO2_, and h_WOO3_ represent heights determined using individual sensors and Δh_surfr_, Δh_WOO2_, and Δh_WOO3_ represent the differences between the heights from the sensors and the reference height h_vg_ from videogrammetry. 

**Figure 11:**
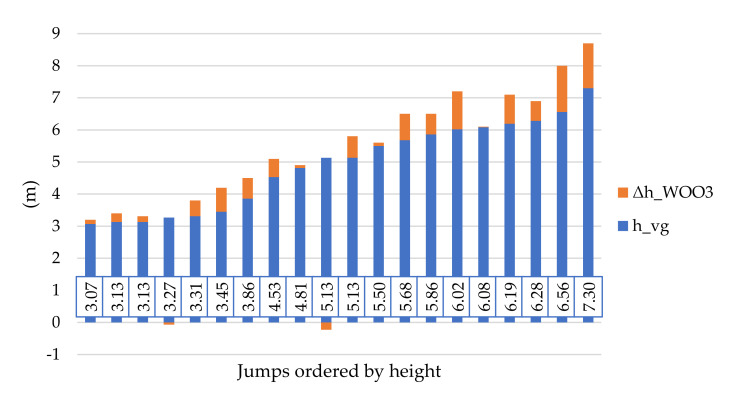
Jump heights determined videogrammetrically and corresponding differences related to WOO3 sensor.

The resulting differences suggest that the most relevant values are provided by the Surfr system and the least reliable by WOO2. Surfr overestimated the jump height at 15, WOO2 at 19, and WOO3 at 18 out of a total of 20 jumps. Even if we considered a tolerance of 0.2 m due to the variable wave height, Surfr overestimated the jump height at 11, WOO2 at 18, and WOO3 at 13 jumps. However, the functional principle of sensors, especially accelerometers, implies that the total error gradually increases with the number of measurements. Therefore, it can be expected that the higher the jump, the greater the difference from the videogrammetric reference value (Figure 11). 

**Figure 12:**
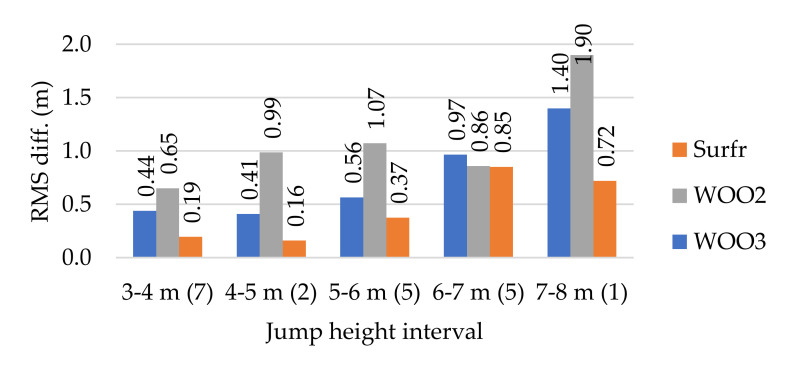
Accuracy of wearable sensors according to jump height. The number of jumps in each group is given in parentheses after the height interval.

This assumption was confirmed for all sensors by dividing the jumps into groups according to the jump height and calculating the RMS of the achieved differences inside of each group (Figure 12).

The smallest differences occurred with the Surfr application installed on the Apple iPhone SE 2016 (model A1723). Up to a height of 5 m, the accuracy was approximately 0.2 m, which roughly corresponded to the height of the waves. However, at greater jump heights, accuracy decreased to approximately 0.4–1 m. The WOO2 sensor proved to be the least accurate (0.6–1.9 m). The WOO3 sensor achieved an accuracy of 0.4 m for jumps up to 5 m. However, for jumps exceeding 5 m, differences were in the range of 0.5–1.4 m.

## 5. Discussion and Conclusions

Videogrammetric measurement of spherical targets mounted on the kiteboard made it possible to determine the reference heights of 20 jumps with an accuracy of 0.03–0.09 m, depending on the distance of the kiter from the cameras. However, when compared to commercial systems such as Surfr, WOO2, and WOO3, variations in the estimated jump height were related to the determination of the zero jump level (owing to a variable wave height) in addition to the accuracy of the videogrammetry and wearable sensors. With small jumps and estimation differences up to 0.2 m it is not possible to reliably determine which of the systems is more accurate. However, jump heights equal to 3 m are of no competitive interest. The Surfr system achieved the best results with the Apple iPhone SE 2016 (model A1723), while the WOO2 sensor produced the least accurate height estimates. Additionally, results show that with increasing jump height, the differences in estimated height based upon the wearable sensors compared to the videogrammetric measurement increased up to 25% of the actual jump height (WOO2, Jump 16). The data obtained also indicated that all of the sensors used tended to overestimate jump height. With the Surfr system, the height was overestimated in 75% of jumps. WOO2 overestimated up to 95% of the jumps and WOO3 overestimated 90% of the jumps. Therefore, the estimated jump heights determined by these sensors (e.g., WOO3 Leaderboards) should be considered as approximations. For jumps with heights exceeding 5 m, the deviations can reach 15–20% of the actual height, therefore jump heights nearing 30 m may show an artificial increase of 4.5–6 m.

All systems that use accelerometers to determine the height of the jump suffer from the negative effects of their technological principle: high frequency leads to a large number of measurements in which various errors accumulate and the resulting value can gradually deviate significantly from reality. To definitively determine the reliability of wearable sensors, it would therefore be appropriate to make a comparison with a reference measurement of approximately 100 jumps of different heights. Especially for the group of higher jumps, which are also competitively more interesting, more jumps would be required. Unfortunately, owing to the prevailing weather conditions in Slovakia, this was not possible. However, despite the smaller sample of jumps in this study, our results confirm the assumptions of the sports community and indicate the approximate achievable accuracy of the tested systems. If additional data become available, the results will be published in a separate article.

## Figures and Tables

**Table 1:** Elements of interior orientation for individual cameras.

Camera	1	2	3	4
f (mm)	24.6636	24.5528	24.6860	24.6552
w (mm/pix)	16.2106/3840	16.2016/3840	16.2074/3840	16.2127/3840
h (mm/pix)	9.1164/2160	9.1164/2160	9.1164/2160	9.1164/2160
xp (mm)	8.2801	8.1329	8.2464	8.0842
yp (mm)	4.6736	4.6419	4.6041	4.6207
K1	0.0001067000	0.0001110000	0.0000962900	0.0001109000
K2	−0.0000003008	−0.0000005818	−0.0000000926	−0.0000000978
K3	0.0000000011	0.0000000031	−0.0000000004	−0.0000000009
P1	−0.0000798300	−0.0000202600	−0.0000756200	0.0000023480
P2	0.0000146600	0.0000187600	−0.0000093620	0.0000081680
P (mm)	0.0042	0.0042	0.0042	0.0042

**Table 2:** Comparison of jump heights determined videogrammetrically and using wearable sensors.

Jump #	h_vg_ (m)	σ_h_ (m)	h_surfr_ (m)	h_WOO2_ (m)	h_WOO3_ (m)	Δh_surfr_ (m)	Δh_WOO2_ (m)	Δh_WOO3_ (m)
1	6.08	0.064	6.90	6.90	6.10	0.82	0.82	0.02
2	6.28	0.063	7.70	7.20	6.90	1.42	0.92	0.62
3	3.31	0.049	3.10	3.80	3.80	–0.21	0.49	0.49
4	6.56	0.036	7.08	7.00	8.00	0.52	0.44	1.44
5	5.68	0.061	6.06	7.00	6.50	0.38	1.32	0.82
6	6.02	0.061	6.83	7.10	7.20	0.81	1.08	1.18
7	3.45	0.064	3.79	4.00	4.20	0.34	0.55	0.75
8	6.19	0.050	6.27	7.10	7.10	0.08	0.91	0.91
9	5.13	0.047	5.18	6.10	4.90	0.05	0.97	−0.23
10	5.13	0.060	4.75	5.80	5.80	−0.38	0.67	0.67
11	3.13	0.055	3.32	3.50	3.40	0.19	0.37	0.27
12	3.13	0.032	3.06	3.20	3.30	−0.06	0.08	0.18
13	5.86	0.054	6.43	7.40	6.50	0.57	1.54	0.64
14	3.27	0.062	3.22	3.20	3.20	−0.05	−0.07	−0.07
15	4.81	0.066	5.02	5.70	4.90	0.21	0.89	0.09
16	7.30	0.089	8.02	9.20	8.70	0.72	1.90	1.40
17	4.53	0.060	4.61	5.60	5.10	0.08	1.07	0.57
18	3.86	0.053	4.10	5.30	4.50	0.24	1.44	0.64
19	3.07	0.045	3.02	3.50	3.20	−0.05	0.43	0.13
20	5.50	0.059	5.81	6.00	5.60	0.31	0.50	0.10
					RMS:	0.51	0.95	0.70
					Max:	1.42	1.90	1.44
					Min:	–0.38	–0.07	–0.23

## Data Availability

Not applicable.
